# pDC Activation by TLR7/8 Ligand CL097 Compared to TLR7 Ligand IMQ or TLR9 Ligand CpG

**DOI:** 10.1155/2019/1749803

**Published:** 2019-04-09

**Authors:** Jing Wu, Shuang Li, Tete Li, Xinping Lv, Mingyou Zhang, Guoxia Zang, Chong Qi, Yong-Jun Liu, Liang Xu, Jingtao Chen

**Affiliations:** ^1^Institute of Translational Medicine, The First Hospital, Jilin University, Changchun 130061, China; ^2^Department of Cardiovascular Center, The First Hospital, Jilin University, Changchun 130031, China; ^3^Sanofi Research and Development, Cambridge, MA 02139, USA; ^4^State key Laboratory of Toxicology and Medical Countermeasures, Beijing Institute of Pharmacology, Beijing 100850, China

## Abstract

Plasmacytoid dendritic cells (pDCs) express high levels of the toll-like receptors (TLRs) TLR7 and TLR9. In response to TLR7 and TLR9 ligands, pDCs are primary producers of type I interferons. Our previous study demonstrated that pDCs activated by the TLR7 ligand imiquimod (IMQ) and the TLR9 ligand CpG A can kill breast cancer cells *in vitro* and inhibit tumor growth in vivo. Moreover, we observed a distinctive morphological, phenotypic change in pDCs after activation by IMQ and CpG A. However, the effect of other TLR7 and TLR9 ligands on pDCs remains less understood. In this study, we treat pDCs with the TLR7 ligand IMQ, TLR7/8 ligands (CL097 and CL075), and three TLR9 ligands (different types of CpGs). The size of pDCs increased significantly after activation by TLR7, or TLR7/8 ligands. TLR7, TLR7/8, and TLR9 ligands similarly modulated cytokine release, as well as protein expression of pDC markers, costimulatory molecules, and cytotoxic molecules. Interestingly, TLR7/8 ligands, especially CL097, induced stronger responses. These results are relevant to the further study of the role and mechanism of pDC-induced antitumor effects and may aid in the development of a new strategy for future tumor immunotherapy.

## 1. Introduction

As antigen-presenting cells, pDCs play an essential role in bridging innate and adaptive immunity [1, 2]. Various ligands and their corresponding receptors are responsible for the signaling events involved in pDC activation and maturation. Natural TLR agonists including uridine-rich ssRNA (ligand of TLR7), and oligodeoxynucleotides (ODNs) with CpG motifs (ligand of TLR9) [3, 4], have shown the capacity to elicit an innate immune response in pDCs. In response to TLR7 and TLR9 ligands, pDCs are the main type 1 interferon- (IFN-) producing cells [5, 6] and thus play a key role in antitumor responses [7, 8]. Several synthetic imidazoquinoline-like molecules, exemplified by imiquimod (IMQ), have been identified as TLR7 ligands and can activate the NF-*κ*B pathway [9]. Interestingly, the TLR7 ligand, imidazoquinoline compound CL097, and the thiazoloquinolone derivative CL075 have also been identified as ligands of TLR8 [10].

Previously, our research demonstrated that pDCs activated by the TLR7 ligand IMQ and the TLR9 ligand CpG A can kill breast cancer cells *in vitro* and inhibit tumor growth *in vivo* [11]. The antitumor response of IMQ-activated pDCs was stronger than that of CpG A-activated pDCs. Besides, we observed a distinctive morphological and phenotypic change in pDCs after activation by IMQ or CpG A. However, the functional mechanisms of other TLR7 and TLR9 ligands, especially TLR7/8 ligands, remain less understood. Therefore, the objective of this study was to compare the effects on pDC activation by different TLR7, TLR7/8, and TLR9 ligands by examining changes in PDC protein expression and cytokine release.

## 2. Materials and Methods

### 2.1. Materials and Reagents

TLR7 ligand IMQ, TLR7/8 ligand CL097, and CL075 were purchased from Invivogen (San Diego, USA). The TLR9 ligands CpG ODN 2216 (5′-GGGGGACGATCGTCGGGGGG-3′) (class A CpG), CpG ODN 2006 (5′-TCGTCGTTTTGTCGTTTTGTCGTT-3′) (class B CpG), and CpG ODN 2395 (5′-TCGTCGTTTTCGGCGCGCGCCG-3′) (class C CpG) were obtained from Sangon (Shanghai, China). An expression vector encoding full-length murine Flt3L cDNA (pORF-mFlt3L) was purchased from Invitrogen (San Diego, USA). Fluorochrome-conjugated anti-mouse antibodies were obtained from BD Biosciences (New Jersey, USA), BioLegend (San Diego, USA), and eBioscience (San Diego, USA). The medium for culturing pDCs was RPMI 1640 (Gibco, Carlsbad, USA) supplemented with 10% fetal bovine serum (Gibco, Carlsbad, USA), 1% penicillin and streptomycin, nonessential amino acids, sodium pyruvate, and *β*-mercaptoethanol, all from Invitrogen (USA).

### 2.2. Mice

Female BALB/c mice were purchased from Vital River Laboratory Animal Technology Co. (Beijing, China) and were maintained in a pathogen-free animal facility at the Institute of Translational Medicine, The First Hospital, Jilin University. Our experiments were performed on 8-10-week-old mice, and all animal experiments were performed according to protocols approved by the Institutional Animal Care and Use Committee at the University of Jilin.

### 2.3. Isolation and Culture of pDCs

Murine pDCs were isolated from the bone marrow of Flt3L-treated mice. Injection of pORF-mFlt3L was performed using the hydrodynamic-based gene delivery technique, as previously described [12]. Ten micrograms of pORF-mFlt3L dissolved in 2 ml of saline was rapidly injected into the tail vein of mice. Bone marrow cells were isolated from femurs and tibiae 10 days after Flt3L plasmid injection, incubated with rat anti-CD16/32 mAbs to block nonspecific binding, and then stained with the following mAbs: anti-CD11c, anti-B220, and anti-CD11b (BD Biosciences). Sorting of pDCs with a CD11c^int^CD11b^–^B220^+^ phenotype was carried out using a BD FACSAria cell sorter (BD Biosciences, USA). The purity of the isolated pDC population was generally >90%. Cell viability determined by trypan blue staining was >99% after isolation. The percentage of pDCs in bone marrow leukocytes is less than 0.4% in naïve mice and is about 2.5% in Flt3L plasmid-injected mice. We can get about 5 × 10^5^ pDCs from each Flt3L plasmid-injected mouse. Cultured pDCs were at a density of 2.5 × 10^6^ cells/ml in RPMI 1640 medium supplemented with 10% FCS, 1% Pen/Strep, nonessential amino acids, sodium pyruvate, and *β*-mercaptoethanol. After activation with TLR7 and TLR9 ligands for 48 hours, pDCs were harvested and assessed for morphological changes by Giemsa staining and for phenotypic changes by flow cytometry.

### 2.4. Giemsa Staining

For Giemsa staining, sorted pDCs from murine bone marrow were cultured in RPMI 1640 medium supplemented with various TLR7 and TLR9 ligands. Cell suspensions were placed on glass slides in a cytospin rotor with a plastic chamber and paper divider at 10^5^ cells per spot. Centrifugation was carried out at 500 × *g* for 10 minutes. Slides were air-dried, fixed in methanol, and stained with modified Giemsa stain GS500 (Sigma Diagnostics, USA). Each of the slide specimens was observed under a light microscope (BX51N-34-FL-1-D, Olympus Corporation, Tokyo, Japan).

### 2.5. Flow Cytometric Analysis

After activation with TLR7 and TLR9 ligands for 24, 48, or 72 hours, pDCs were harvested and stained with the following mAbs: anti-CD11c (557401), anti-CD11b (550993), anti-B220 (553092), anti-CD40 (553791), anti-CD80 (560523), and anti-PD-L1 (558091) all from BD Biosciences; anti-MHC class II (107605) and anti-CD86 (105031) from BioLegend; and anti-BST2 (25-3172-82), anti-Siglec-H (11-0333-82), and anti-Granzyme B (11-8898-82) all from eBioscience. Intracellular staining was performed using a cell permeabilization kit (BD Biosciences). After incubation with the respective Abs for 25 minutes at 4°C, cells were washed twice and then subjected to flow cytometric analysis. Mean fluorescence intensity (MFI) values of Ab staining, after subtraction of the MFI of the respective isotype, were depicted in FACS plots.

### 2.6. ELISA and CBA Assays

We cultured pDCs at 2.5 × 10^6^ cells/ml with TLR7 and TLR9 ligands in RPMI medium supplemented with 10% FCS, 1% Pen/Strep, nonessential amino acids, sodium pyruvate, and *β*-mercaptoethanol. Supernatants were collected after 36 hours and analyzed with ELISA (IFN-*α* and Granzyme B; R&D Systems, USA) and CBA (TNF-*α*, IL-12p70, IL-6, and IL-10; BD Pharmingen, USA). All ELISA results are expressed in pg/ml. For CBA assays, 50 *μ*l of samples or recombinant standards was added to 50 *μ*l mixed capture beads and incubated for 3 hours with 50 *μ*l of phycoerythrin-conjugated detection antibodies (Ab-PE) to form sandwich complexes. After washing samples to remove the unbound Ab-PE reagent and acquiring them on a flow cytometer (FACSArray, BD, USA), data was generated using FCAP Array software.

### 2.7. Statistical Analysis

Data were analyzed using GraphPad Prism software and expressed as means ± standard error of the mean (SEM). Statistical differences were calculated using a one-way analysis of variance (ANOVA), followed by Tukey test for multiple comparisons. Statistical significance was inferred where *p* < 0.05.

## 3. Results

### 3.1. Morphological Changes to pDCs after Activation with Ligands of TLR7, TLR7/8, and TLR9

**Figure 1:**
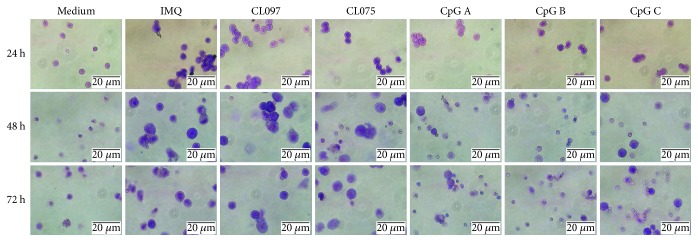
Ligands of TLR7 can induce a dramatic morphological change in pDCs. We harvested pDCs after activation with TLR7 ligands (IMQ, CL097, and CL075) and TLR9 ligands (CpG A, CpG B, and CpG C) for 24, 48, and 72 hours. The concentration for each ligand was 1.5 *μ*M. We then assessed pDCs for morphological changes by Giemsa staining. Representative images from one of three experiments were shown. For each stained section, at least three images from random fields were taken. Magnification was ×500 (oil immersion).

We established the optimal concentration of TLR7 and TLR9 ligands to activate pDCs in a previous study [11]. This earlier work showed that 1.5 *μ*M of IMQ and CpG was optimal for induction of MHC II, CD40, CD80, and CD86. Hence, we used 1.5 *μ*M of TLR7, TLR7/8, and TLR9 ligands in this study. We cultured pDCs with TLR7, TLR7/8, and TLR9 ligands and performed Giemsa staining 24, 48, and 72 hours after coculture. Our results showed that, compared with medium, IMQ, CL097, and CL075 can induce a dramatic morphological change in pDCs. The cells were enlarged significantly, with cell diameter expanding two- to threefold compared with the medium control. This morphological change was observed as early as 24 hours after stimulation, was even more evident at 48 hours, and was still present at 72 hours. Similar changes were not induced by CpG A, CpG B, or CpG C at the indicated time points (Figure 1).

### 3.2. Expression of Markers on pDCs after Activation with Ligands of TLR7, TLR7/8, and TLR9

**Figure 2:**
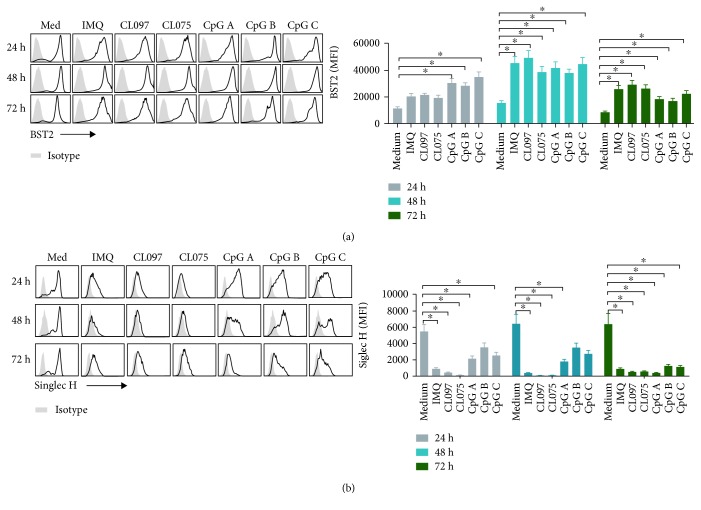
The expression of markers on pDCs after activation with TLR7 and TLR9 ligands. We harvested pDCs after activation with TLR7 ligands (IMQ, CL097, and CL075) and TLR9 ligands (CpG A, CpG B, and CpG C) for 24, 48, and 72 hours. The concentration of each ligand was 1.5 *μ*M. We then assessed the expression of markers BST2 (a) and Siglec-H (b) on pDCs by flow cytometry. Data shown are expressed as mean ± SEM (*n* = 6/group) and represent three independent experiments with similar results. Statistical significance of differences between groups was determined; ^∗^ *p* < 0.05.

BST2 and Siglec-H are two additional important markers of pDCs, so we further measured the expression of BST2 and Siglec-H in pDCs before and after stimulation with different TLR7, TLR7/8, and TLR9 ligands. Before stimulation, BST2 and Siglec H-positive pDCs are more than 90% (Figure S1). Compared with medium, pDCs cocultured with CpG A, CpG B, or CpG C had significantly higher expression of BST2 at 24 hours (Figure 2(a)). At 48 hours, pDCs cocultured with the TLR7 and TLR9 ligands IMQ, CL097, CL075, CpG A, CpG B, and CpG C had a significantly increased expression of BST2 (Figure 2(a)). We observed similar changes at 72 hours after coculture (Figure 2(a)). Siglec-H expression on pDCs was significantly decreased compared with that in the medium at 24, 48, and 72 hours after coculture with the TLR7 and TLR9 ligands IMQ, CL097, CL075, CpG A, CpG B, or CpG C (Figure 2(b)). This effect was stronger for TLR7 and TLR7/8 ligands than for TLR9 ligands.

### 3.3. The Expression of Costimulators on pDCs after Stimulation with TLR7, TLR7/8, and TLR9 Ligands

**Figure 3:**
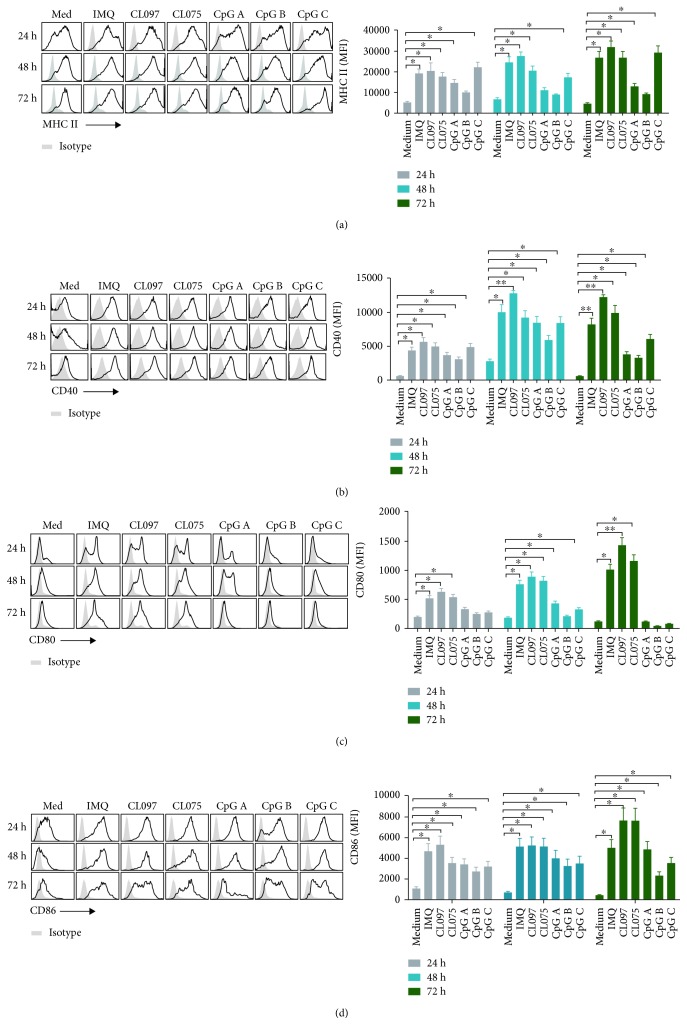
Ligands of TLR7, especially CL097, can upregulate the expression of costimulators on pDCs. We harvested pDCs after activation with TLR7 ligands (IMQ, CL097, and CL075) and TLR9 ligands (CpG A, CpG B, and CpG C) for 24, 48, and 72 hours. The concentration of each ligand was 1.5 *μ*M. We then assessed the expression of costimulators MHC-II (a), CD40 (b), CD80 (c), and CD86 (d) on pDCs by flow cytometry. Data shown are expressed as mean ± SEM (*n* = 3-6/group) and represent three independent experiments with similar results. Statistical significance of differences between groups was determined; ^∗^ *p* < 0.05 and ^∗∗^ *p* < 0.01.

As antigen-presenting cells, pDCs express costimulatory molecules MHC class II, CD40, CD80, and CD86 and play the role of antigen presentation. We further measured the expression of costimulators of pDCs after stimulation with different TLR7, TLR7/8, and TLR9 ligands. At 24, 48, and 72 hours after coculture with the TLR7, TLR7/8, and TLR9 ligands IMQ, CL097, CL075, CpG A, CpG B, and CpG C, MHC-II expression on pDCs was significantly higher than that in the medium (Figure 3(a)). We observed a similar change in CD40 expression after coculture with TLR7, TLR7/8, and TLR9 ligands, where the effect of CL097 was most evident (Figure 3(b)). The TLR7 and TLR7/8 ligands IMQ, CL097, and CL075 significantly elevated the expression of CD80 on pDCs at 24, 48, and 72 hours after coculture. CpG A and CpG C also elevated CD80 expression on pDCs at 48 hours after coculture, but at other time points there was no change in CD80 expression after stimulation with TLR9 ligands (Figure 3(c)). We also observed a significantly elevated expression of CD86 at 24, 48, and 72 hours after coculture with the TLR7, TLR7/8, and TLR9 ligands (Figure 3(d)).

### 3.4. The Expression of Cytotoxic Molecules on pDCs after Activation with Ligands of TLR7, TLR7/8, and TLR9

**Figure 4:**
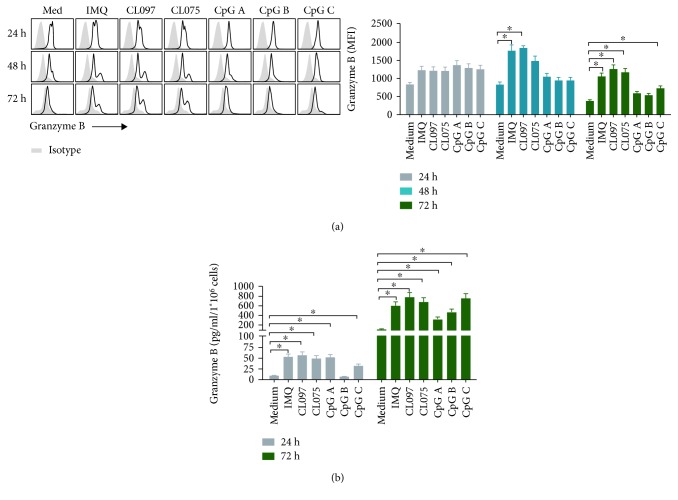
Ligands of TLR7, especially CL097, can upregulate the expression of cytotoxic molecules on pDCs. pDCs and supernatants were harvested after activation with TLR7 ligands (IMQ, CL097, and CL075) and TLR9 ligands (CpG A, CpG B, and CpG C) for 24, 48, and 72 hours. The concentration of each ligand was 1.5 *μ*M. We then assessed the expression of cytotoxic molecules Granzyme B (a, b) on pDCs by flow cytometry and ELISA. Data shown are expressed as mean ± SEM (*n* = 3-6/group) and represent three independent experiments with similar results. Statistical significance of differences between groups was determined; ^∗^ *p* < 0.05.

Next, cytolytic molecules Granzyme B expressed by TLR7 and TLR9 ligands stimulating pDCs were determined. The results showed that expression of MFI of Granzyme B was significantly higher on pDCs at 48 hours after coculture with IMQ and CL097 and at 72 hours after coculture with IMQ, CL097, CL075, and CpG C (Figure 4(a)). Granzyme B concentration was significantly higher 24 hours after coculture with ligands of TLR7, TLR7/8, and TLR9, with the exception of CpG B, and was raised 72 hours after coculture with all ligands (Figure 4(b)). The values significantly increase in pDC medium control supernatants at 72 hours. This may be due to substantial cell death in the medium control conditions without any stimulation. When some cells are not active and disintegrate, Granzyme B is released to the medium, significantly increasing the values in this group.

### 3.5. Expression of Immune-Suppressive Molecules on pDCs after Activation with Ligands of TLR7, TLR7/8, and TLR9

**Figure 5:**
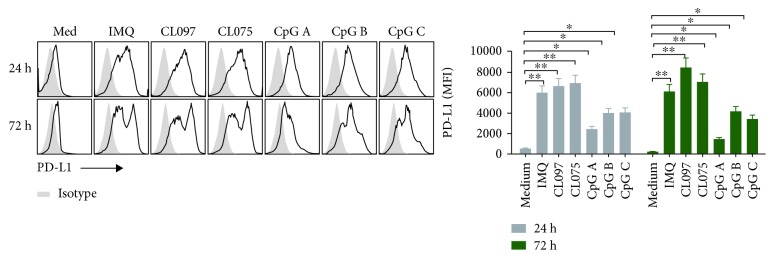
Ligands of TLR7 and TLR9 can significantly increase PD-L1 expression on pDCs, and this function is stronger for TLR7 ligands than for TLR9 ligands. We harvested pDCs after activation with TLR7 ligands (IMQ, CL097, and CL075) and TLR9 ligands (CpG A, CpG B, and CpG C) for 24, 48, and 72 hours. The concentration of each ligand was 1.5 *μ*M. We then assessed the expression of the immune-inhibitory molecule PD-L1 on pDCs by flow cytometry. Data shown are expressed as mean ± SEM (*n* = 3/group) and represent three independent experiments with similar results. Statistical significance of differences between groups was determined; ^∗^ *p* < 0.05 and ^∗∗^ *p* < 0.01.

When pDCs are either unstimulated or alternatively activated, they express programmed death-ligand 1 (PD-L1) and promote tolerance to tumor cells. Therefore, we measured PD-L1 expression after stimulation by the TLR7, TLR7/8, and TLR9 ligands. Compared with controls, we found that all TLR7, TLR7/8, and TLR9 ligands tested can significantly increase PD-L1 expression at both 24 and 72 hours after coculture. This effect was stronger for TLR7 and TLR7/8 ligands than for TLR9 ligands (Figure 5).

### 3.6. The Release of IFN-*α*/TNF-*α*/IL12p70/IL-6 from pDCs after Stimulation with TLR7, TLR7/8, and TLR9 Ligands

**Figure 6:**
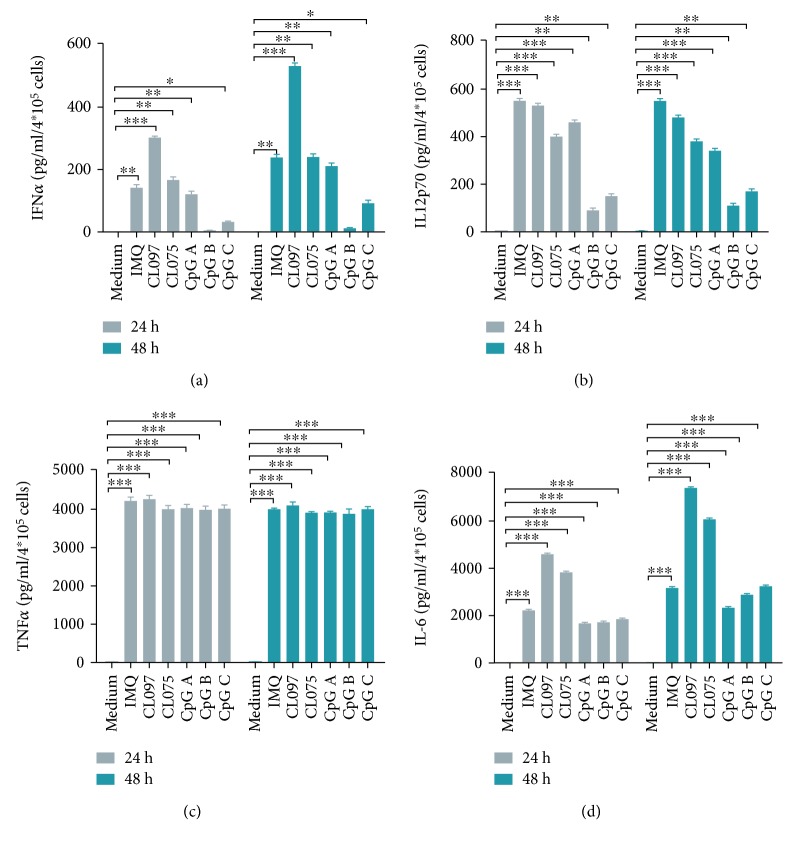
Ligands of TLR7 and TLR9 can significantly increase the release of IFN-*α*/TNF-*α*/IL12p70/IL-6 from pDCs. This effect is stronger for TLR7 ligands than for TLR9 ligands. Supernatants from the pDC culture medium were harvested after pDC activation with TLR7 ligands (IMQ, CL097, and CL075) and TLR9 ligands (CpG A, CpG B, and CpG C) for 24 and 48 hours. The concentration of each ligand was 1.5 *μ*M. Cytokine release was assessed by ELISA and CBA. Data shown are expressed as mean ± SEM (*n* = 6/group) and represent three independent experiments with similar results. Statistical significance of differences between groups was determined; ^∗^ *p* < 0.05, ^∗∗^ *p* < 0.01, and ^∗∗∗^ *p* < 0.001.

Cytokine release after pDC activation plays a critical role in the immune response. We measured concentrations of IFN-*α*, IL12p70, TNF-*α*, and IL-6 after stimulation with ligands of TLR7, TLR7/8, and TLR9 (Figure 6). We found that the release of IFN-*α* from pDCs was increased significantly after stimulation with ligands of TLR7 and TLR7/8 (IMQ, CL097, and CL075) and ligands of TLR9 (CpG A and CpG C). This effect was observed at both 24 and 48 hours but was absent for CpG B. The effect was the strongest for CL097 (Figure 6(a)). Compared with medium control, the concentrations of cytokines IL12p70, TNF-*α*, and IL-6 were significantly increased from pDCs at both 24 and 48 hours after stimulation with TLR7, TLR7/8, and TLR9 ligands, and the effect of CL097 was consistent among the strongest (Figures 6(b)–6(d)).

## 4. Discussion

The crucial role of pDCs in antiviral responses involves sensing viral infection through TLR7 and TLR9, which results in the production of large amounts of IFN-I [5]. As a bridge to interface the innate and adaptive immune responses, pDCs can trigger the activation and differentiation of natural killer (NK) cells, myeloid dendritic cells, B cells, and T cells. In addition, pDCs have been shown to mediate tolerance to cardiac allografts, oral antigens, and airway antigens, which is consistent with their antigen presentation capabilities [13, 14]. pDCs are rather inhibited or modulated by the tumor microenvironment, which has been described as a mechanism to escape immunosurveillance and maintain an immunosuppressive tumor microenvironment [15–19]. However, it has been shown that pDCs activated by the TLR7 ligand IMQ or the TLR9 ligand CpG were capable of initiating effective antitumor immunity through the activation of NK cells, mDCs, and CD8^+^ T cells in a mouse melanoma model, as well as in melanoma patients [7, 8, 20–22]. Our previous research demonstrated that IMQ- and CpG A-activated pDCs could kill breast cancer cells in vitro and inhibit breast tumor growth in vivo, at which IMQ was more effective than CpG A. However, the effect on pDCs of other TLR7 and TLR9 ligands is less well understood.

The results presented here demonstrate that the size of pDCs increased significantly after activation by TLR7 ligands, while no such change was observed after TLR9 ligand activation (Figure 1). The significant enlargement of pDCs may be indicative of their activation and functional change. In addition, TLR7 and TLR9 ligands similarly modulated the protein expression of the pDC markers BST2 and Siglec-H (Figure 2); its costimulatory molecules MHC II, CD40, CD80, and CD86 (Figure 3); and its cytotoxic molecules Granzyme B (Figure 4). They also modulated release by pDCs of the cytokines IFN-*α*, IL-12p70, TNF-*α*, and IL-6 (Figure 6). Overall, these effects were stronger for TLR7 ligands than for TLR9, especially CL097. These results will help guide the choice of adjuvants in the future.

In addition, ligands of TLR7 and TLR9 can upregulate the expression of the immune-inhibitory molecule PD-L1, and this is consistent with recent reports [23]. The effect of TLR7 ligands is also stronger than TLR9 ligands in this role (Figure 5). These phenomena may reflect negative regulation of immunity to maintain the immune balance of the body. Our observations may therefore provide more choices for immunotherapeutic targets against tumors and autoimmune disease.

Activation of TLR7 and TLR9 receptors in pDCs results in secretion of type I IFNs (IFN-I) via the MyD88-IRF7 pathway as well as production of proinflammatory cytokines via the MyD88-NF-*κ*B pathway [24, 25]. Besides the MyD88 pathway, additional factors determine whether TLR9 signaling engagement will result in IFN-I or proinflammatory cytokine (e.g., IL-6, IL-12, and TNF-*α*) production. CpG A is transported to the IRF7 and NF-*κ*B endosome and is a strong inducer of IFN-I and proinflammatory cytokine, while CpG B aggregates in the endosome and is a potent stimulator of cytokine production. CpG C has the characteristics of both CpG A and CpG B in that it can induce both IFN-I and proinflammatory cytokines. TLR7 stimulation by viral and endogenous RNA may follow similar pathways. Our data showed that release of IFN-*α* from pDCs was increased significantly after stimulation with ligands of TLR7 and TLR7/8 (IMQ, CL097, and CL075) and ligands of TLR9 (CpG A and CpG C), but was absent for CpG B; compared with medium control, the concentrations of cytokines IL12p70, TNF-*α*, and IL-6 were significantly increased from pDCs at both 24 and 48 hours after stimulation with TLR7, TLR7/8, and TLR9 ligands (Figures 6(b)–6(d)). This effect was stronger for TLR7 and TLR7/8 ligands than for TLR9 ligands, especially CL097 (Figure 6).

Besides proinflammatory cytokines IL12p70, TNF-*α*, and IL-6, we also measured a tolerogenic cytokine IL-10 released from activated pDCs. Few IL-10 were detected after pDC activation by CpG B and CpG C (Figure S2). It builds on the characterization of TLR7/8-actvated pDCs as more proinflammatory than TLR9-activated pDCs.

However, there is no clear standard to screen promising adjuvant, whether IFN-I or proinflammatory cytokines productions or whether molecular changes. At present, functional assays and even in vivo tests may be needed to explore the potential exerting direct or immune-mediated antitumor effects. Thus, it will be critical to address these questions in future studies.

In our experimental results, the function of CL097 was stronger than that of IMQ. Synthetic imidazoquinoline-like molecules exemplified by IMQ have been identified as ligands of TLR7. The imidazoquinoline compound CL097 and the thiazoloquinolone derivative CL075 are preferential ligands of TLR7 and TLR8, respectively [9, 26]. It has been reported that pDCs contribute to skin fibrosis in systemic sclerosis largely through activation of TLR8 [27]. Additionally, vaccinia virus DNA could induce TLR8-mediated activation of murine pDCs [28]. This may reflect a stronger activation of pDCs by CL097, at least in comparison with IMQ, as seen in our results. However, the function of CL075 is similar to IMQ, so the specific reasons are not very clear and are a subject for future exploration.

Loschko et al. [29] have shown that the recognition of antigens by Siglec-H inhibits pDC-induced adaptive responses. Siglec-H was defined as a specific marker for pDCs, the expression of which can distinguish between proinflammatory and tolerogenic pDCs [29]. In our experiment, Siglec-H expression on pDCs significantly decreased after activation with TLR7 and TLR9 ligands, with the effect of TLR7 ligands being much stronger than the effect of TLR9 ligands (Figure 2(b)). Consistent with these results, a lower expression of Siglec-H on activated pDCs correlated with reduced presence of immunosuppressive cells and cytokines that facilitate tumor development.

## 5. Conclusions

Our study revealed that pDCs could be directly activated by the TLR7 ligand IMQ and by the TLR7/8 ligands CL097 and CL075. In addition, pDCs could be activated by the TLR9 ligands CpG A, B, and C in a similar mechanism. Interestingly, the effects of the TLR7 ligands tested, especially the TLR7/8 ligand CL097, were stronger than those of TLR9 ligands. The results in this report therefore support the potential utility of the TLR7/8 ligands CL097 and CL075, the TLR7 ligand IMQ, and the TLR9 agonists CpG A, B, and C, in the treatment of cancer or other diseases in which the activation of pDCs may be desirable. Further functional assays will facilitate the development of approaches to choose the most promising adjuvant.

## Data Availability

The data used to support the findings of this study are included within the article.

## Supplementary Materials

Supplementary Materials Figure S1: the expression of BST2 and Siglec H on sorted pDCs before stimulation. Figure S2: the release of IL-10 from pDCs after stimulation with TLR7, TLR7/8, and TLR9 ligands.
