# Supplementary figures and images for: pDC Activation by TLR7/8 Ligand CL097 Compared to TLR7 Ligand IMQ or TLR9 Ligand CpG
Source: J Immunol Res. 2019 Apr 9;2019:1749803. doi: 10.1155/2019/1749803 (PMC6481147; doi:10.1155/2019/1749803)

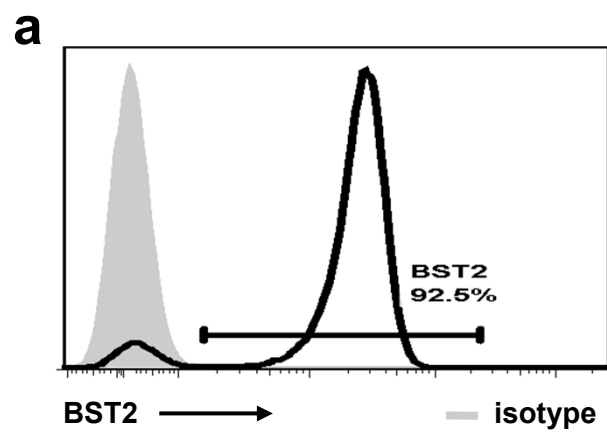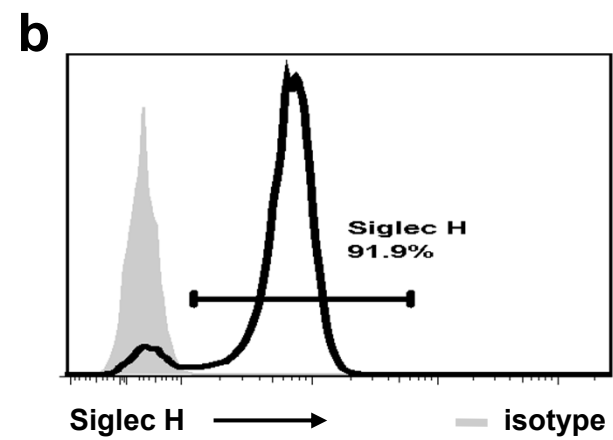

**Figure S1**

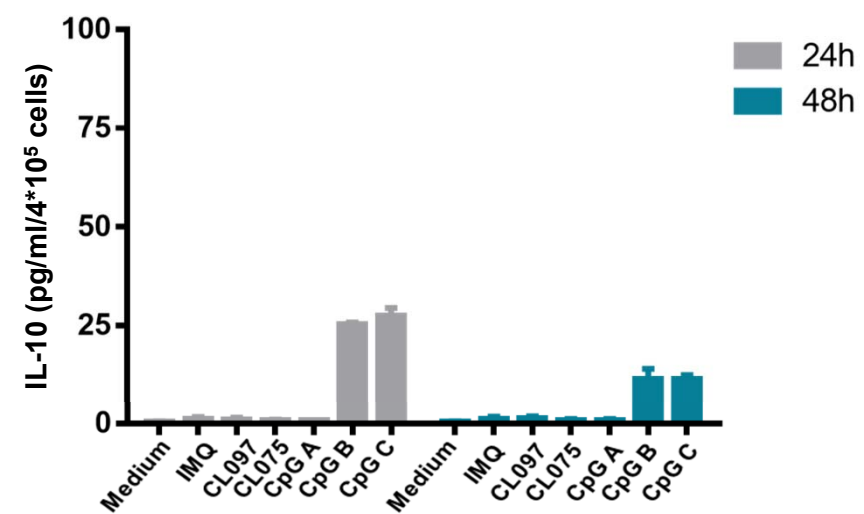

**Figure S2**

Supplement: Supplementary Materials — Figure S1: the expression of BST2 and Siglec H on sorted pDCs before stimulation. Figure S2: the release of IL-10 from pDCs after stimulation with TLR7, TLR7/8, and TLR9 ligands. [file 1749803.f1.pdf]
